# Reinforced thinned-skull window for repeated imaging of the neonatal mouse brain

**DOI:** 10.1117/1.NPh.9.3.031918

**Published:** 2022-06-03

**Authors:** Vanessa Coelho-Santos, Taryn Tieu, Andy Y. Shih

**Affiliations:** a Seattle Children’s Research Institute, Center for Developmental Biology and Regenerative Medicine, Seattle, Washington, United States; b University of Washington, Department of Pediatrics, Seattle, Washington, United States; c University of Washington, Department of Bioengineering, Seattle, Washington, United States

**Keywords:** cranial window, cerebrovascular, neurodevelopment, neurovascular unit, live imaging, two-photon microscopy

## Abstract

**Significance:**

Two-photon microscopy is a powerful tool for *in vivo* imaging of the mammalian brain at cellular to subcellular resolution. However, resources that describe methods for imaging live newborn mice have remained sparse.

**Aim:**

We describe a non-invasive cranial window procedure for longitudinal imaging of neonatal mice.

**Approach:**

We demonstrate construction of the cranial window by iterative shaving of the calvarium of P0 to P12 mouse pups. We use the edge of a syringe needle and scalpel blades to thin the bone to ∼15-μm thickness. The window is then reinforced with cyanoacrylate glue and a coverslip to promote stability and optical access for at least a week. The head cap also includes a light-weight aluminum flange for head-fixation during imaging.

**Results:**

The resulting chronic thinned-skull window enables *in vivo* imaging to a typical cortical depth of ∼200  μm without disruption of the intracranial environment. We highlight techniques to measure vascular structure and blood flow during development, including use of intravenous tracers and transgenic mice to label the blood plasma and vascular cell types, respectively.

**Conclusions:**

This protocol enables direct visualization of the developing neurogliovascular unit in the live neonatal brain during both normal and pathological states.

## 1 Introduction

The brain represents only ∼2% of the body’s total mass yet consumes ∼20% of its resting energy production. This high demand for energy and the lack of substantial energy reserve make the brain heavily reliant on continuous blood flow through a vascular network. Several features of the brain vasculature must be established in early stages of postnatal life, including (1) the formation of a dense and highly interconnected capillary network for blood distribution,^1^^,^^2^ (2) a blood–brain barrier (BBB) that is highly selective to solute and macromolecule transport,^3^ and (3) proper regulation of neurovascular coupling to match the metabolic demands of neural tissue to increased supply of oxygenated blood.^4^^,^^5^

The development and maturation of the microvasculature involves a complex interplay between endothelial cells with nearly all other brain cell types. This process is orchestrated throughout embryogenesis and the initial weeks after birth,^6^ where extensive cerebrovascular growth and remodeling occurs. Pericytes, astrocytes, microglia, and neurons all interact with brain endothelial cells to shape the structure and function of the vascular network, and the importance of these cellular relationships is emphasized by a conceptual framework called the neurogliovascular unit (NVU).^6^ The complex interactions within the NVU promote, regulate, and maintain the functions of the BBB and establish pathways involved in neurovascular coupling.

Despite many crucial advances in our understanding of cerebrovascular development and BBB maturation,^6^^,^^7^ the complex cellular dynamics involved in construction of the cerebrovasculature remain poorly understood. This gap in knowledge is partly due to the dearth of techniques for longitudinal, high-resolution brain imaging of developing mammals. *In vivo* imaging of very early brain development can be performed with relative ease in lower vertebrates, such as fish and chicks, over the full temporal range of vascular growth and remodeling. Imaging of mouse embryos in utero is difficult. However, the development of the capillary network and BBB integrity in mice continues into the postnatal period, making it accessible for imaging by two-photon microscopy.^2^^,^^6^^,^^8^^,^^9^ Chronic imaging windows allow the dynamics of biological processes to be tracked over time, providing insight beyond that gathered by conventional histological approaches. Further, chronic imaging for mice *in vivo* is desirable because of the availability of diverse fluorescent transgenic lines that allow NVU cell types to be visualized.

**Fig. 1:**
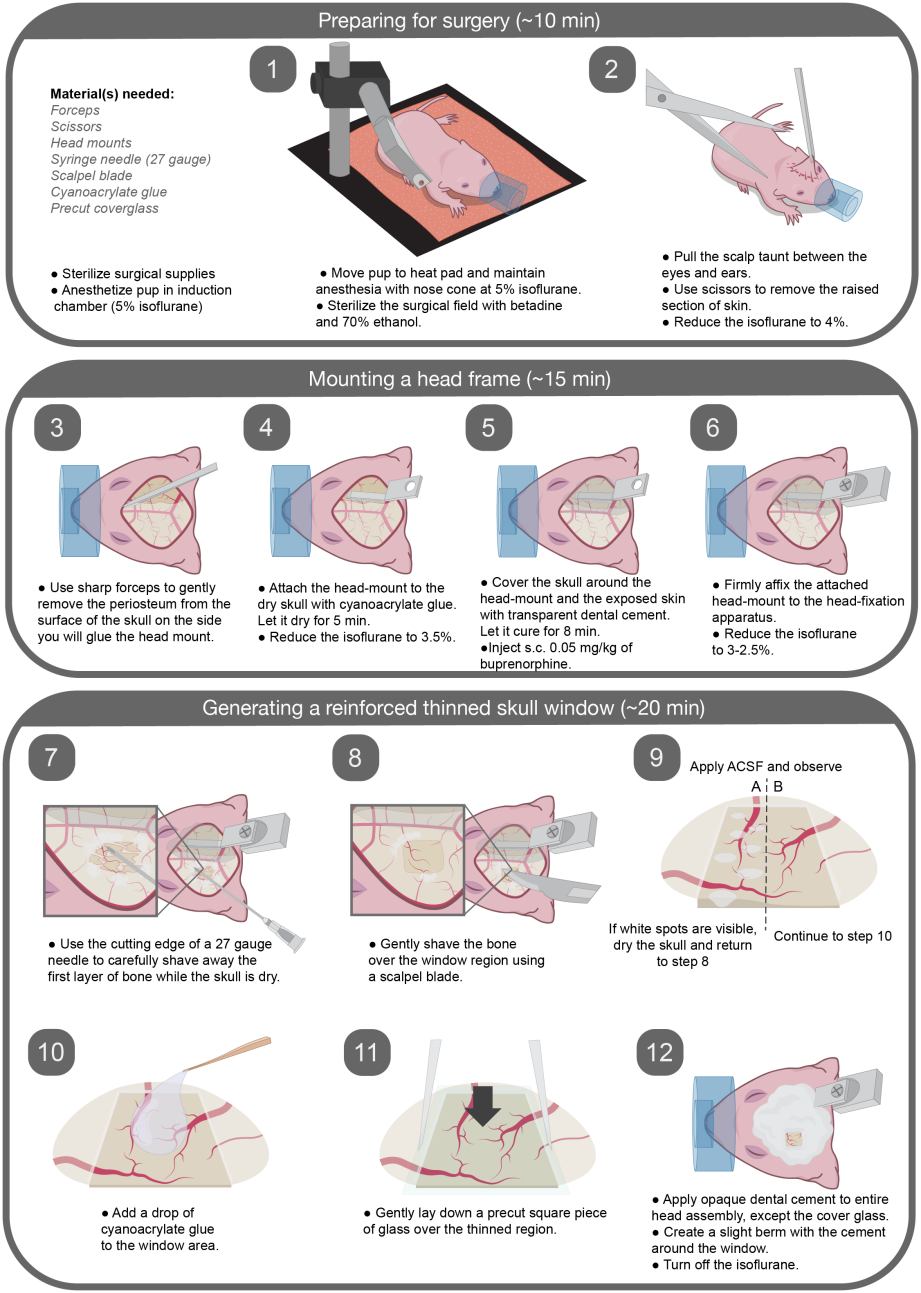
Graphical description of all surgical steps.

To our knowledge, only two published studies have used *in vivo* two-photon imaging to study mouse cerebrovascular development at the microvascular level.^9^^,^^10^ Both studies were cognizant of the spurious effects of skull removal on vascular function and therefore used thinned-skull windows to minimize disturbance of the intracranial environment. This process involves delicately shaving of the skull until it is sufficiently thin for optical access. However, in one study, the window was not a chronic implant, and required repeated surgeries and re-thinning of the skull.^9^ The second study^10^ used chronic thinned skull windows, but the windows were made using a burr drill, which may promote vibration-induced tissue damage. Here, we build on these protocols by thinning the skull with the cutting edge of syringe needles and scalpel blades to avoid mechanical vibration (Fig. 1). We then apply cyanoacrylate glue and a cover glass to reinforce the stability of the window. Using this neonatal reinforced thin-skull window, the cortical vasculature was imaged as early as P0 for acute experiments and longitudinally between P7 to P12,^2^ which encompasses an extensive period of capillary growth and remodeling in the cerebral cortex. Unraveling the coordination of NVU components during development is an exciting challenge and we believe this method will contribute to advances in the field.

## 2 Protocol

### 2.1 Materials and Reagents

#### Reagents

1. Animal tattoo ink paste (Ketchum Mfg., 329A)
2. Fluorescent dextran dye of high molecular weight:
  a. Tetramethylrhodamine (TMR)–dextran 2MDa [Thermo, D7139; 2.5% w/v in phosphate-buffered saline (PBS)]
  b. Fluorescein isothiocyanate (FITC)–dextran 2MDa (Sigma-Aldrich, FD2000S; 2.5% w/v in PBS)
  c. Alexa 680 2MDa (custom conjugated as described;^11^ 5% w/v in PBS).
3. Betadine solution (Patterson Veterinary, 07-808-8855)
4. Buprenorphine hydrochloride (Patterson Veterinary, 07-893-3796)
5. Ethanol, 70% v/v in water (Fisher Scientific, A405F-1GAL)
6. Isoflurane (Patterson Veterinary, 07-893-1389)
7. Lactated Ringer’s solution (Fisher Scientific, AAJ67572AP)
8. Sterile artificial cerebral spinal fluid. We used a modified artificial cerebral spinal fluid^12^ (125-mM NaCl, 10-mM glucose, 10-mM HEPES, 3.1-mM ${\mathrm{CaCl}}_{2}$, 1.3-mM MgCl_2_, pH 7.4) (all chemicals from Sigma-Aldrich).

#### Disposables

1. Air can duster (Amazon, Falcon Dust-Off, B07QJY6MKD)
2. Cotton-tipped applicators (Fisher Scientific, 23-400-100)
3. Cyanoacrylate glue (Amazon, Loctite Instant Adhesive 401, B006GOKRSY)
4. Dental cement kit (Parkell, C&B-METABOND, S380). Specific reagents used include the following:
  a. “C” Universal TBB Catalyst (Parkell, C&B-METABOND, S371)
  b. Metabond “B” quickbase (Parkell, C&B-METABOND, S398)
  c. Adjustable Precision Applicator Brushes (Parkell, C&B-METABOND, S379)
  d. Radiopaque L-Powder (Parkell, C&B-METABOND, S396)
  e. Clear L-Powder (Parkell, C&B-METABOND, S399).
5. Kimwipes (Kimberly Clark, Kimtech, 34133)
6. Cover glass (precut with Tip Scriber pen to 2 to $4\;{\mathrm{mm}}^{2}$ area, Electron Microscopy Sciences Slide Cover Glass #0, 22×22  mm, Ref 7219810)
7. Ringer solution (Fisher Scientific, AAJ67572AP)
8. Surgical scalpel blade (Fisher Scientific, #15, 22-079-701)
9. Insulin syringe, 0.3 cc (VWR, BD328438)
10. Syringe needles (27 and 31 gauge, BD Bioscience, 305109)

#### Surgical and other small tools

**Fig. 2:**
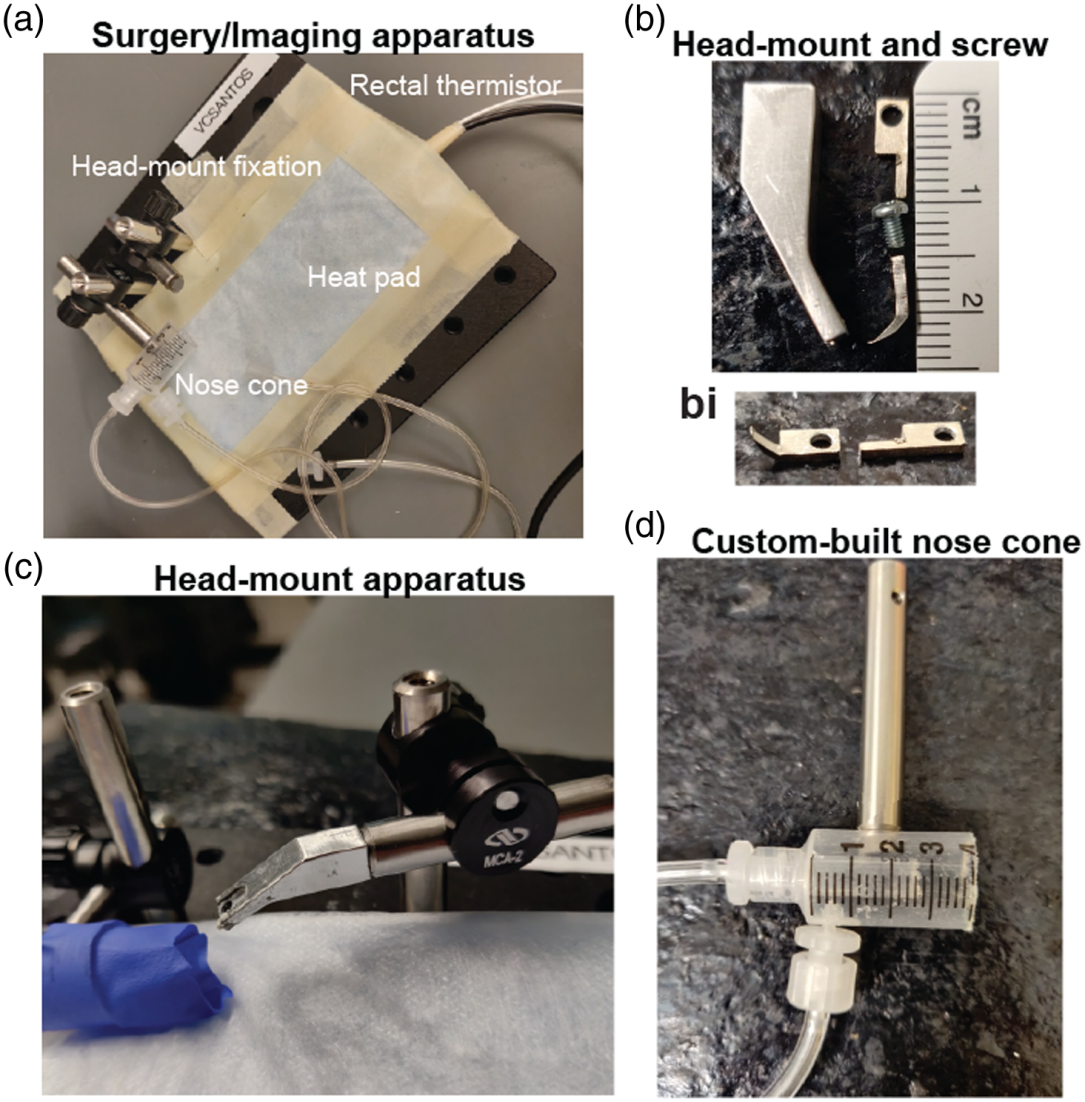
Custom components for window implantation and imaging. (a) Apparatus used for surgery and imaging. (b) Custom aluminum holding arm and head mount with screw for head fixation. (bi) The metal flange to be adhered to the skull is ∼0.04  g in weight and should be bent and shaped before use. (c) Head-fixation apparatus from a side view. (d) Custom-made nose cone for isoflurane delivery. One line of tubing is for isoflurane input, and the second is for vacuum scavenging.

1. Aluminum head mount flange and holder [Figs. 2(a) and 2(b)], custom-built

(Figures S1 and S2 in the Supplemental Materials, Solidworks plans: Ref. 13)

Steel Pan Head Phillips Screws (McMaster Carr, 90272A040)

The aluminum flange needs to be bent and shaped prior to use [Fig. 2(bi)].

2. Forceps, Dumont no. 55 (Fine Science Tools, 11255-20)
3. Forceps, serrated (Fine Science Tools, 11050-10)
4. Head-fixation apparatus [Fig. 2c]:

Thorlabs: Breadboard 6×6 MB6; adapter with external 8-32 threads and external 1/4″-20 threads (AP8E25E).

Newport: Adjustable-angle post clamp, 8-mm diameter optical posts, MODEL: M-MCA-2; 8-mm diameter optical posts TSP2.

5. Nose cone [Fig. 2(d), we used a custom-made nose cone, but commercial versions can also be used]
6. Screwdriver, miniature (Garret Wade, 26B09.01)
7. Surgical scissors, blunt end (Fine Science Tools, 14078-10).

#### Equipment

1. Dissecting microscope (Olympus SZX10 or equivalent)
2. Glass bead sterilizer (Braintree Scientific, Inc., Germinator 500)
3. Feedback regulated temperature control (FHC Inc., 40-90-8D – DC) with heat pad (FHC Inc., 40-90-2) and rectal thermistor for mouse (FHC Inc., 40-90-5D-02)
4. Isoflurane vaporizer (IsoTec4; Datex-Ohmeda, GE Healthcare)
5. 20-W LED fiber optic illuminator, dual gooseneck lights or equivalent (Dolan-Jenner, Fiber-Lite Mi-LED, 16103-DG)
6. Laser power meter (Thor Labs, PM100D)
7. Two-photon microscope [we used a Bruker Investigator 2 photon microscope with Spectra-Physics Insight X3 laser, but similar instruments from other companies can be used. Emission bandpass filters included 460/40  nm (blue), 525/70  nm (green), 595/50  nm (red), and 660/40  nm (far red)] and 20-X, 1.0 NA objective lens (Olympus XLUMPLFLN 20XW).

#### Software

1. Fiji/ImageJ
2. Matlab.

### 2.2 Step-by-Step Methods Details

**Fig. 3:**
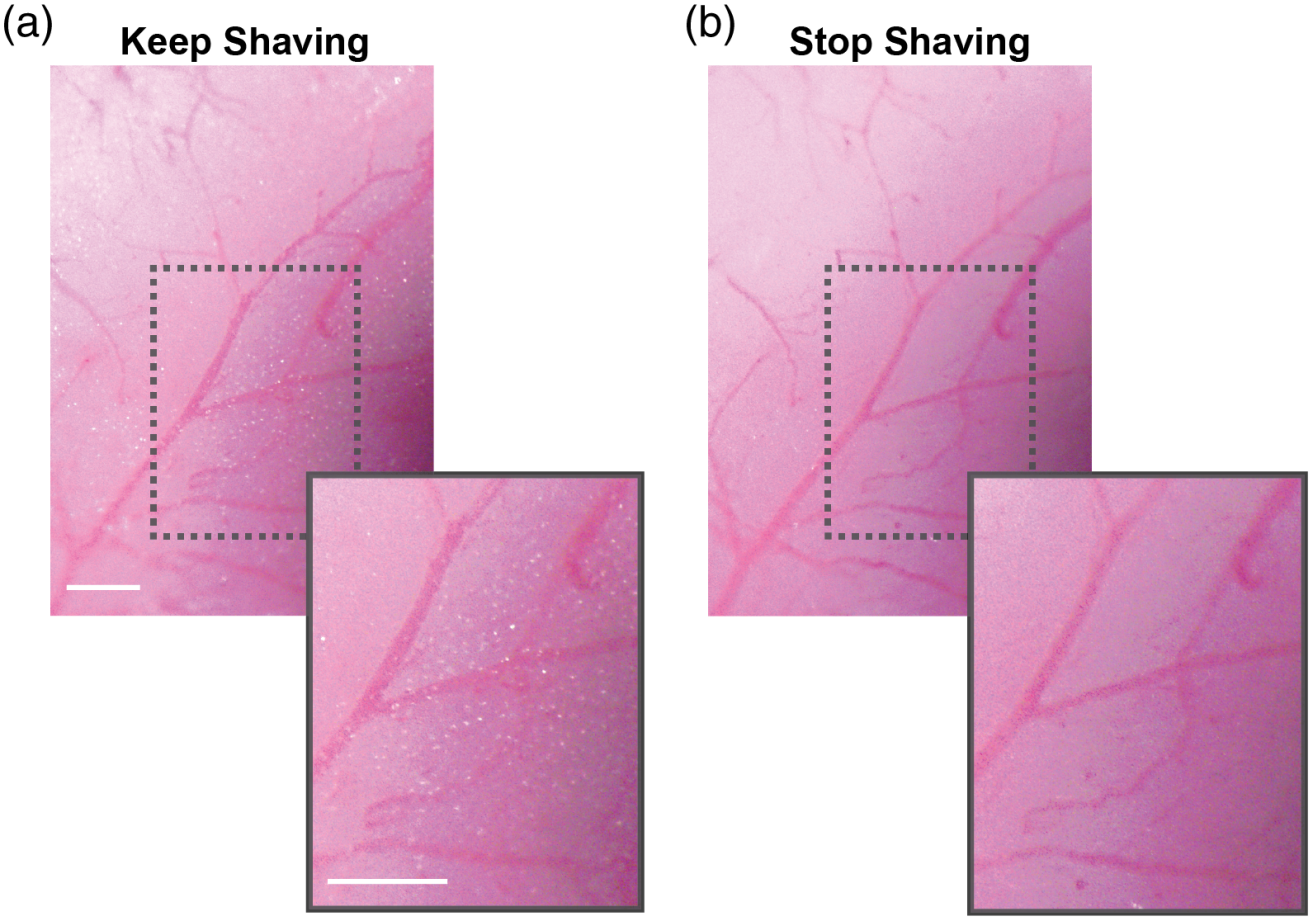
Examining the thinned-skull window for translucency. Examples show (a) when the skull should be shaved more, with white dots being seen when the bone is still too thick, and (b) when skull thickness is sufficient to complete the window. Scale bar 1 mm.

**Fig. 4:**
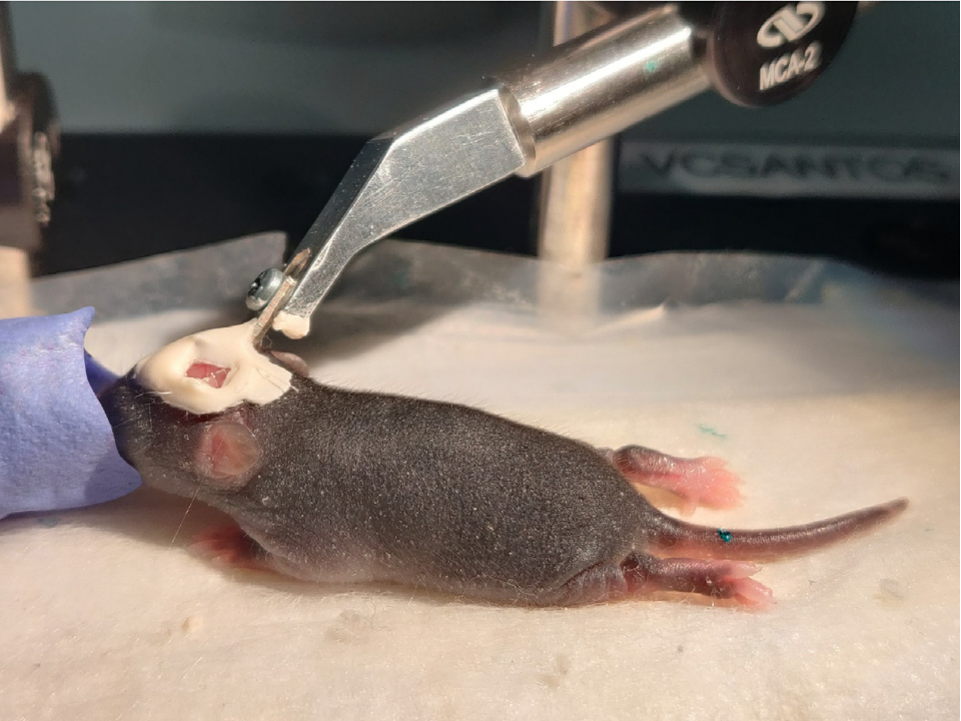
Image of a P7 mouse pup after window implantation and head-fixation on the imaging apparatus.

1. Preparing for surgery. ∼5 to 10 min
  a. Autoclave surgical tools before each surgery. In between animals, use the bead sterilizer to sanitize tools.
  b. Ensure that all necessary reagents and disposables are available. Reagents and disposables that contact exposed tissue should be sterilized.
  c. Anesthetize pups with isoflurane in an induction chamber [about 5% minimum alveolar concentration (MAC) induction in medical oxygen]. Note: some pups are more resistant to isoflurane and may require longer durations of induction.
  d. Place the pup in prone position on a feedback-regulated heat pad to maintain body temperature at 37°C. Maintain anesthesia using isoflurane delivery through the nose cone, reducing it gradually as the surgery progresses, until 2.5% MAC is reached. Note: the rectal thermistor, which is too large for pups, is taped to the heat pad and the temperature is set to 37°C. Insulate the heat pad from the optical breadboard.
  e. Clean the scalp with Betadine followed by 70% ethanol.
2. Mounting a head frame. ∼15  min
  a. Ensure surgical plane of anesthesia by checking for lack of tail pinch reflex. Pull the scalp taunt between the eyes to just caudal of the ears. Then use scissors to cut away the region of scalp lifted from the skull. Reduce the isoflurane to 4%.
  b. Use sharp forceps to gently scrape away the periosteum from the surface of the skull. Since the skull is not fully formed at the age of window implantation, take care to apply minimal force and avoid contact with the skull sutures.
  c. Opposite to the hemisphere of window placement, attach the custom aluminum head mount to the dry skull surface with a thin layer of cyanoacrylate glue for stability during cranial window implantation and subsequent imaging. Let the glue dry for 4 to 5 min. Reduce the isoflurane to 3.5%.
  d. Cover the exposed skin surrounding the skull with transparent dental cement. Then apply the dental cement to the entire assembly, except at the location of window placement (we generally place the windows within the somatosensory cortex, 1- to 4-mm lateral and 0.5- to 2.5-mm posterior to bregma.^14^). Let the cement cure for 7 to 8 min. Note: Metabond powders come in both opaque and transparent forms. We find that the transparent dental cement dries faster, and we use it for portions of the surgery to reduce exposure to anesthesia.
  e. As the cement cures, provide buprenorphine subcutaneously. (0.05  mg/kg, 50-μl volume) mixed with Ringer solution for analgesia and hydration. Note: If multiple pups in a litter are to receive surgery, mark the tails of the pups using a 31 gauge needle and animal permanent tattoo ink to distinguish between them.
  f. Firmly affix the attached head mount to the head-fixation apparatus using a screw. Reduce the isoflurane between 2.5% and 3%. Monitor the breathing frequency.
3. Generating a reinforced thinned skull window. ∼15 to 20 min
  a. Use the cutting edge of a 27 gauge needle to remove the first layer of bone carefully at the site of the window. This is performed while the skull is dry. Position the needle edge at a 10 deg angle from the horizontal, and gently scrape bone away from the skull surface. Angles larger than 10 deg from the horizontal should be avoided because the needle may perforate into the brain. Use gentle flow from the air can duster to remove bone shavings. Note: It is time to switch to a blade when you need to increase the angle to shave more.
  b. After shaving the first layers of bone away with the needle tip, start shaving to achieve a more polished finish using a surgical scalpel blade. A gentle flow of the air from the duster can be used to remove bone shavings. Note: If the vasculature of the bone starts to bleed from any region beyond the window site, use gentle compression with a cotton swab to stop it. If extensive bleeding occurs at the window site, we advise not proceeding with the surgery.
  c. To check if the window is sufficiently translucent, moisten the skull with artificial cerebral spinal fluid. The ideal skull thickness is reached when white dots within the bone are no longer seen immediately after ACSF application (Fig. 3). To dry the skull again prior to shaving, absorb the ACSF with a cotton swab or use gentle air flow from the duster.
  d. Once the thinning process is complete, clean the window area with artificial cerebral spinal fluid, and allow it to dry completely. Use the duster to accelerate the drying process.
  e. Add a drop of cyanoacrylate glue to the window area, carefully avoiding the creation of bubbles, and gently lay down a precut square piece of glass (2 to $4\;{\mathrm{mm}}^{2}$) over the thinned region. Note: Excess glue that covers the glass surface can be removed with a cotton swab. Then allow the glue to dry, and scrape the remaining glue from the glass surface using the cutting edge of a 27 gauge needle.
  f. Apply opaque dental cement to the edges of the cover glass, and create a slight berm with the cement to hold water for the water immersion lens during imaging. Note: Turn off the isoflurane while the cement is drying to decrease total duration of anesthesia.
4. Final steps and critical notes
  a. Steps 1.1 to 3.5 should ideally be completed within 35 to 45 min, as longer surgery times lead to poorer survival.
  b. Following cranial window implantation, rub the pup with the bedding from the home cage to mask any foreign scent and allow the pup to completely recover from anesthesia prior to returning to the home cage.
  c. While the pups recover from anesthesia, introduce small pieces of Metabond dental cement to the cage to allow the dam and sire to adapt to the smell. We find that mouse pups receiving window implantation rapidly acclimate to the head mounts. In each litter, we regularly use several mouse pups for imaging, which helps to reduce the likelihood of one pup being singled out and killed by the dam or sire. If bleeding is observed around the windows or if imaging clarity is lost in the windows, the pups should be excluded from further studies.
5. Preparing for imaging.
  a. Image the pups starting the first day after cranial window implantation. For each imaging session, we advise a maximum time of 1 h. Note: for acute experiments, it is possible to perform live imaging immediately after the surgery with the duration of 1–1.5 h. If longitudinal experiments are desired, better survival is seen when the imaging is started on the day after surgery, or if imaging on the same day as surgery is limited to 30 min or less.
  b. Anesthetize the pups with isoflurane (4% to 5% MAC in medical air) and secure to head-fixation apparatus (Fig. 4).
  c. Inject 10 to 20  μl of a fluorescent dextran dye with a high molecular weight (i.e., 2MDa) through the retro-orbital vein under deep isoflurane anesthesia (4% to 5% MAC) to visualize the cortical microvasculature [for intravenous (i.v.) injection protocol, see Ref. 15]. Using a 0.3-cc insulin syringe is more accurate for small volumes and leads to less waste (no dead volume). Using a high molecular weight dye helps to avoid dye extravasation at early age imaging time points.^2^ It is less problematic to use dyes that are small, i.e., 70 kDa, when pups are >P10. Note: One drawback is i.v. dye retention by cells with mast cell-like appearance at the pial surface.^16^ To minimize accumulation of this unwanted signal, alternate between different dye colors across imaging days. For example, use 2MDa TMR at P8, P10, and P12 and 2MDa Alexa 680 at P9 and P11.
  d. Reduce isoflurane to ∼1.5% MAC in medical air, which will leave the pup anesthetized but reactive to a light toe pinch during imaging.
  e. Gently clean the cranial window surface with a moist cotton swab.
  f. Proceed with two-photon imaging.

## 3 Representative Results

**Fig. 5:**
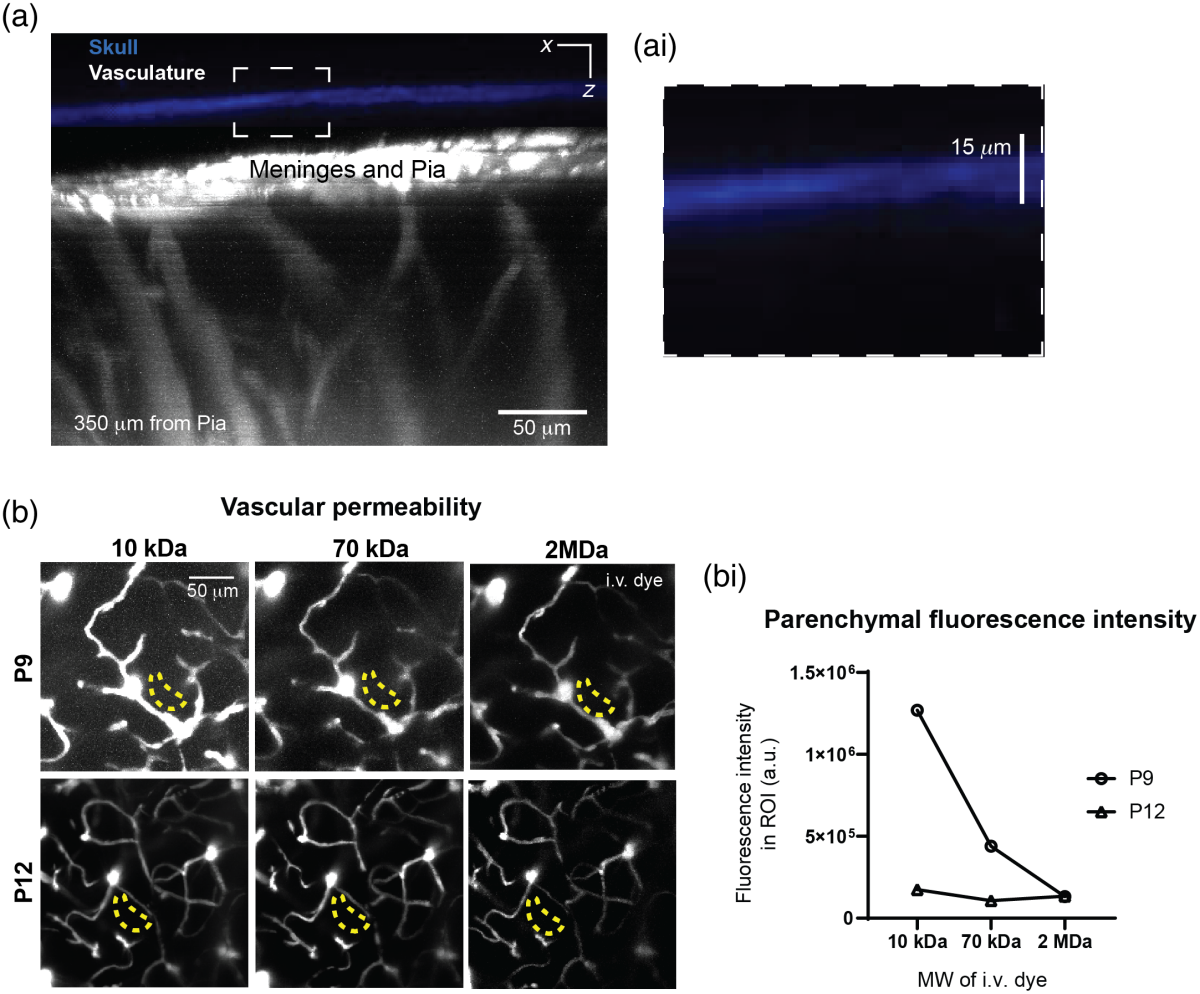
Skull thickness and dye extravasation. (a) Maximally projected side views showing skull thickness and brain vasculature. (ai) The skull (blue), ∼15  μm in thickness, was detected by collecting second harmonic fluorescence. The vasculature (white) was labeled with intravenously injected FITC-dextran. (b) Assessing BBB permeability to fluorescent dextrans of differing molecular weight in the neonate cortex (10-kDa Alexa 647-dextran, 70-kDa Texas Red-dextran, and 2MDa FITC-dextran). (bi) A gradual decrease in permeability is seen between P9 to P12.

To determine the thickness of the skull after completion of the window, we imaged blue second harmonic fluorescence from the bone at 800-nm excitation [Fig. 5(a)]. This revealed that skull thickness was approximately 12 to 15  μm in our preparations [Figs. 5(a) and 5(ai)]. Shaving the skull thinner may lead to breaches in the skull. Although it is not possible to further thin the skull after window completion, it provides a reference for how thin the skull needs to be in future surgeries. As we previously published,^2^ this procedure of thinned skull in neonates does not cause activation of cortical microglia compared with the non-surgery contralateral hemisphere.

Consistent with continued postnatal refinement of the BBB,^6^ we found that some smaller sized dyes leak into the brain parenchyma over time [Fig. 5(b)]. For this reason, improved imaging quality can be achieved with higher MW dyes, including 2.5% 2MDa TMR-dextran, 2.5% 2MDa FITC–dextran, or 5% 2MDa Alexa 680-dextran. Mast cells are developing and colonizing^16^ the brain during P8 to P12 and retain the intravenously injected dyes. Thus, we recommend (if possible) alternating between dyes of the same size but with different fluorophore, as discussed above.^2^

**Fig. 6:**
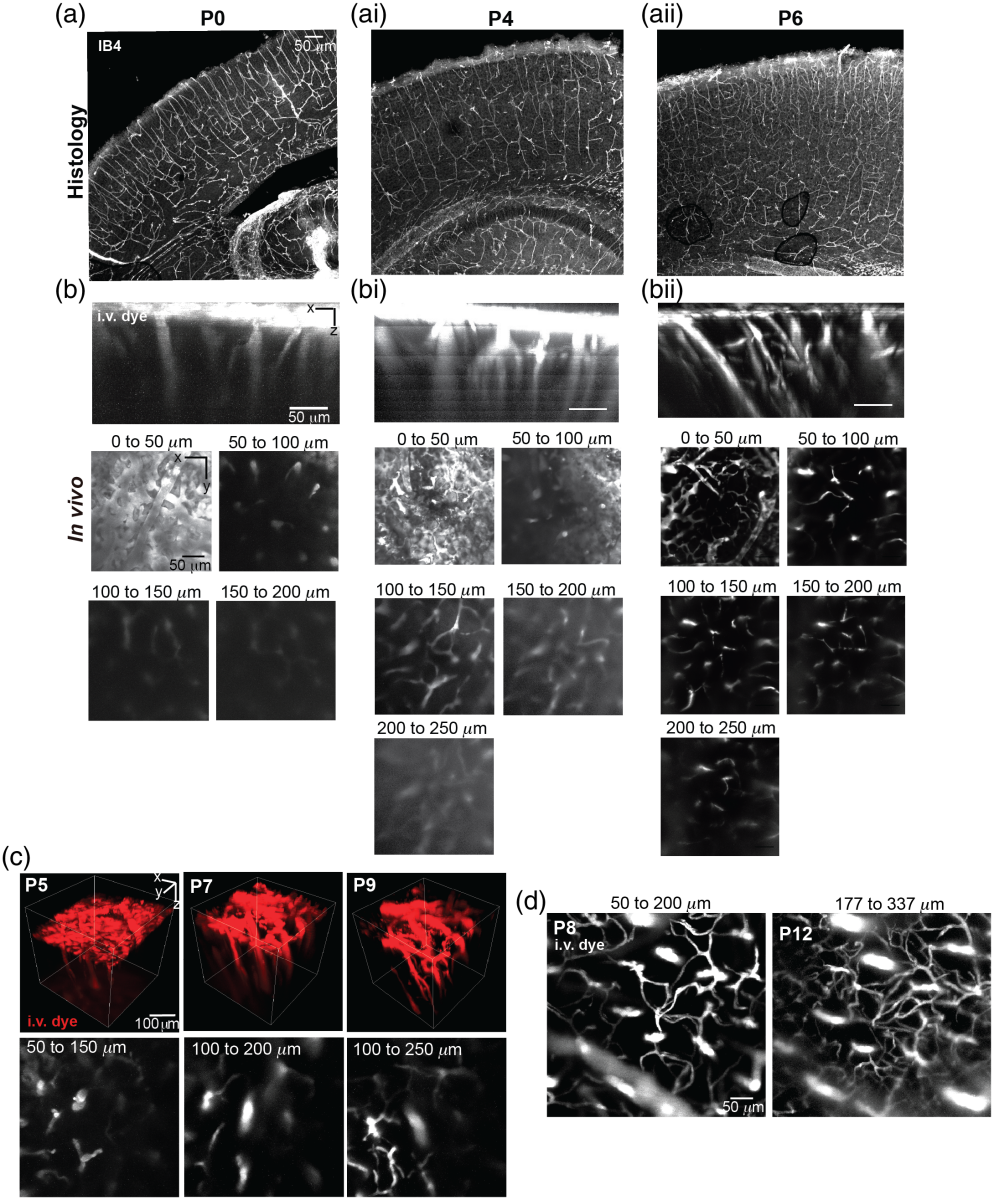
Comparison between two-photon laser-scanning microscopy and histology. Histology of brain slices from (a) P0, (ai) P4, (aii) P6 mouse vessels stained with IB4 lectin. Two-photon image stacks acquired at the same ages of (b) P0, (bi) P4, and (bii) P6, and using 2MDa FITC for the i.v. dye, ∼30-mW laser power, and 800-nm excitation. Maximal projections in X–Y view over 50  μm of tissue taken at different depths below the pia. (c) Longitudinal imaging from P5 to P9. Upper row shows 3D visualization of FITC-labeled mouse brain vasculature. (ci) Images from the same animal as in panel (c) showing projected 150-μm stack at P8 and P12, using Alexa 680 as i.v. dye, ∼50-mW laser power, and 1200-nm excitation.

We were able to generate thinned skull windows across a range of postnatal ages [Fig. 6(a)] and image capillaries within the cortex to a depth of 200 to 250  μm in the brain of mice at P6 to P8 (Fig. 6). However, this depth was difficult to achieve in younger pups from P0 to P4. The achievable imaging depths increases over time as light penetration improves once the pial venular network regresses to cover less of the brain surface.^2^ Under optical conditions, it is possible to image to a depth of 350 to 400  μm in P12 pups (Fig. 6d).

**Fig. 7:**
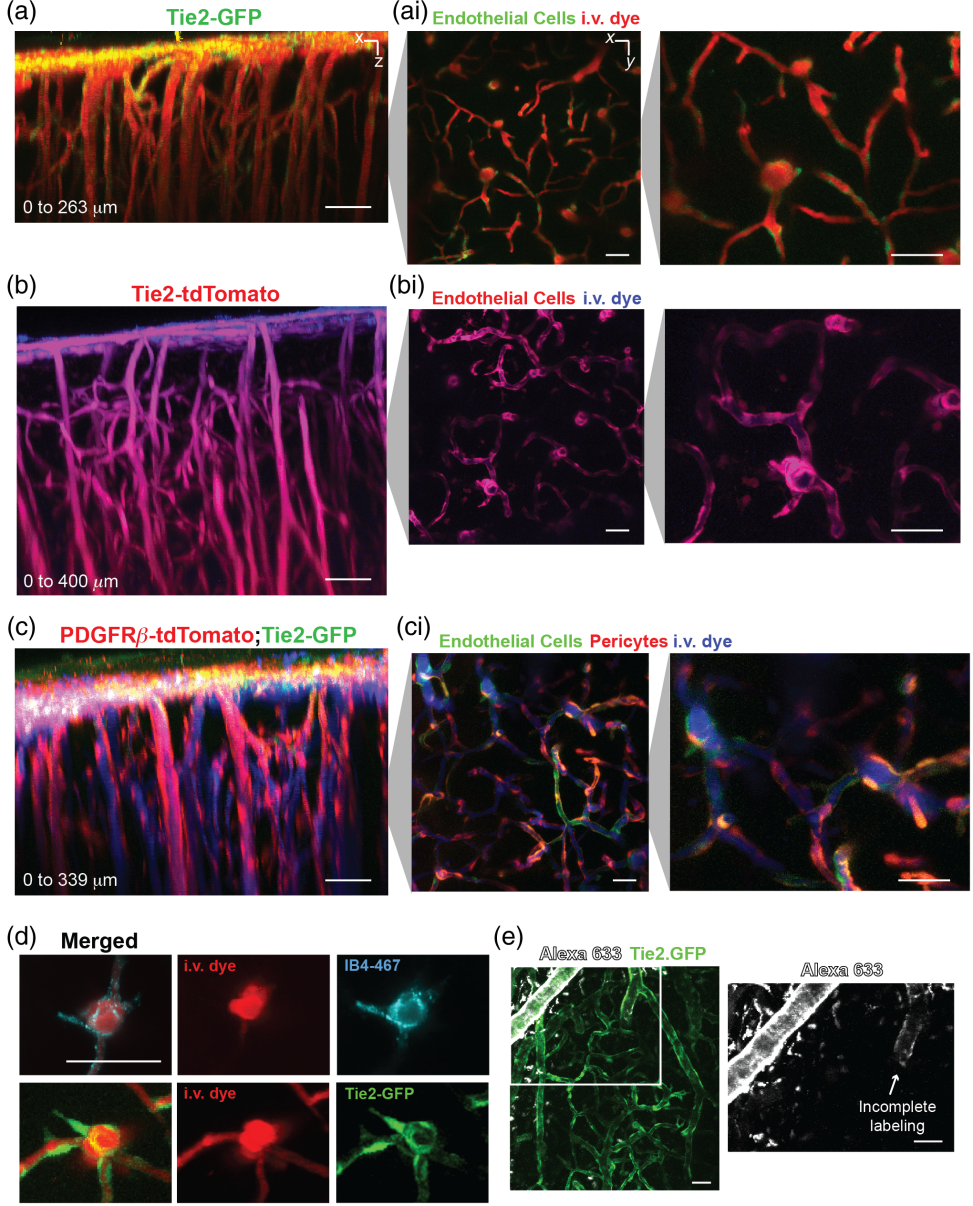
Example transgenic mice and small molecule probes for *in vivo* optical imaging of vasculature at postnatal day 9. (a) Side view of image stack from Tie2-GFP mouse and Alexa 680 as i.v. dye, ∼75-mW laser power, and 920-nm excitation. (ai) Magnified images in X–Y view showing detail of the endothelial cells. (b) Side view of image stack from Tie2-TdTomato mouse and Alexa 680 as i.v. dye, ∼45-mW laser power, and 975-nm excitation. (bi) Magnified images in X–Y showing detail of the endothelial cells (c) Side view of image stack from Tie2-GFP::PDGFR*β*-tdTomato and Alexa 680 as i.v. dye, ∼75-mW laser power, and 975-nm excitation. (ci) Magnified images in X–Y showing detail of the endothelial cells and pericytes. (d) Example of angiogenic sprout with endothelium label by IB4-647 lectin and Alexa 680 as i.v. dye, ∼50-mW laser power, and 1200-nm excitation. (e) Example of pial vasculature with a selective labeling of artery walls by Alexa Fluor 633, ∼40-mW laser power, and 1100-nm excitation. Scale bars=50  μm.

An array of transgenic mice are available to study different NVU cell types.^17^ Some transgenics express fluorescent proteins driven by cell-specific promoters. For example, the widely used Tie2-green fluorescent protein (GFP) mouse has endothelial-specific GFP expression linked to Tie2 promoter activity,^18^ providing an *in vivo* reporter for heightened signaling during vascular remodeling^2^ [Fig. 7(a)]. Alternatively, Cre drivers with specificity for certain neurovascular cell types can be crossed with reporter mice to produce robust fluorescent protein labeling. An example is breeding of constitutive Tie2-Cre (Jax #008863) with Ai14 reporter (Jax #007914) mice to obtain exceptionally bright tdTomato labeling of endothelial cells (and some microglia cells) [Fig. 7(b)]. To study mural-endothelial cell interplay during development, we created triple transgenic mice, Tie2-GFP::PDGFR*β*-Cre::Ai14,^2^ with endothelial cells and pericytes fluorescently labeled in green and red, respectively [Fig. 7(c)].

Fluorescent small molecule probes are also effective for imaging in neonates. For example, we show that IB4-647 can be used for labeling angiogenic sprouts *in vivo* similar to its use in histology.^19^ The dye can take ∼30 to 45 min to label, and fluorescence will persist for several hours [Fig. 7(d)]. Alexa 633 is used in adults to label the elastin in walls of arteries and arterioles.^20^ We find that it can also be used in neonatal preparations, although labeling is dimmer and more incomplete perhaps because the vessel wall is still in an immature state [Fig. 7(e)**]**.

**Fig. 8:**
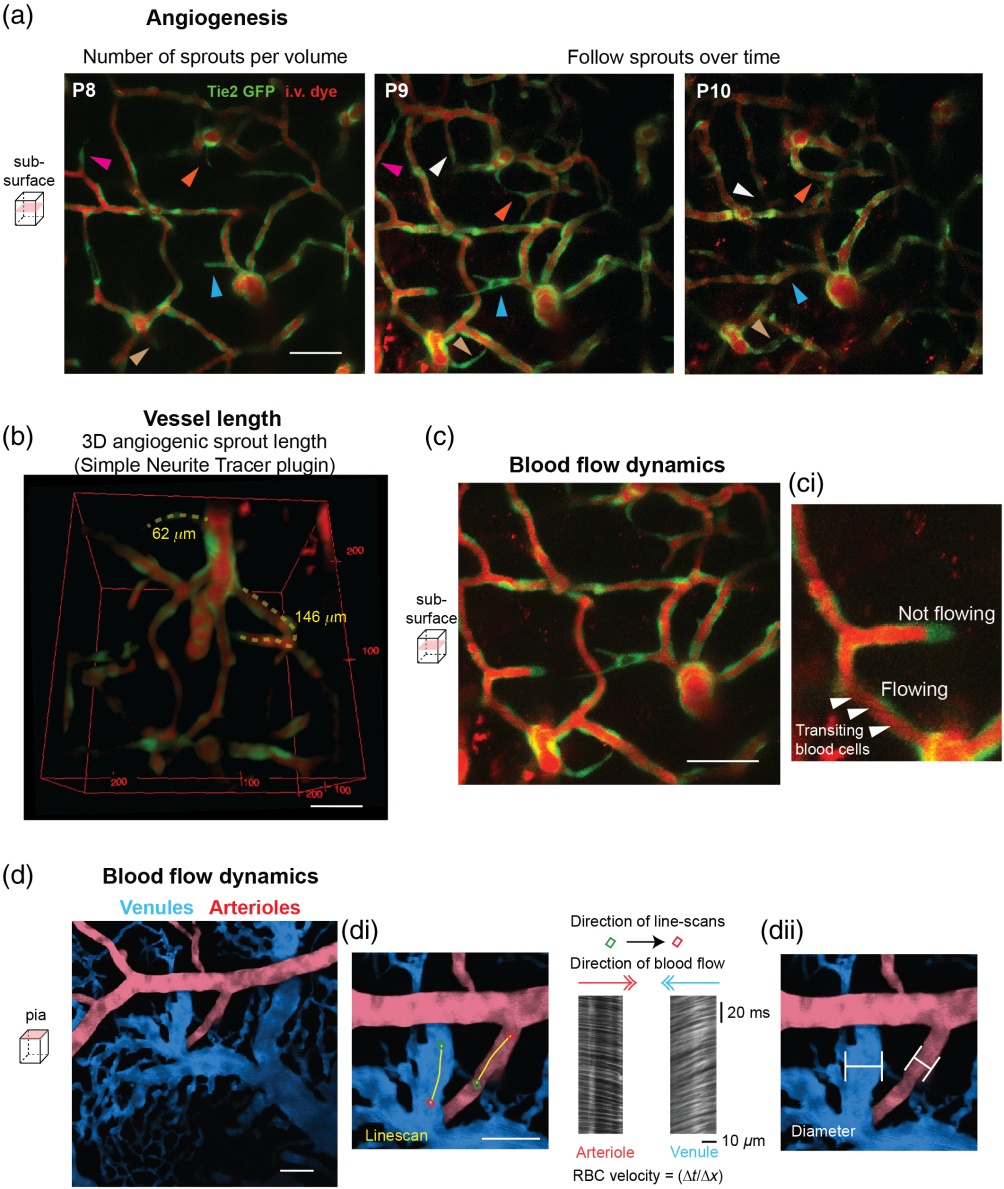
Acquiring metrics of angiogenesis and blood flow with *in vivo* two-photon imaging. (a) Number of angiogenic sprouts on a Tie2-GFP over time (different colored arrowheads represent different angiogenic sprouts, not all are highlighted). I.v. dye alternated between dyes 2MDa Alexa 680 (P9) and tetramethylrhodamine-dextran (P10), ∼70-mW laser power, and 920-nm excitation. (b) Angiogenic sprout length. (c) Blood flow dynamics associated with angiogenic sprouts. (d) Blood flow dynamics in pial arterioles and venules. (di) Direction of blood flow and velocity assessed with line-scanning. (dii) Diameter measurements collected manually in ImageJ/Fiji. Scale bars=50  μm.

The quantification of vascular metrics extracted depend upon the type of cell label and mouse strain. For understanding capillary network formation, we examined angiogenesis by following angiogenic sprouts over time [Fig. 8(a)]. We measured the length of the sprouts [Fig. 8(b)] and the timing at which they become patent [Figs. 8(c) and 8(ci)]. We correlated this information with the overall blood flow collected from the pial vasculature^2^ [Fig. 8(b)]. For analysis of blood cell velocity from line-scan data [Fig. 8(di)], we collected line-scans along the center line of the pial vessel at a rate of ∼1  kHz and used custom Matlab software from Kim et al.^21^ The lumen diameter was calculated using an ImageJ-based macro called VasoMetrics^22^ [Fig. 8(dii)]. Blood cell velocity and vessel diameter was then combined using a formula based on Poiseuille’s law of laminar flow to obtain the volume flux of blood flow, which is a more complete metric of flow through a single vessel.^12^

## 4 Discussion

We have described a method for longitudinal imaging of cerebrovascular development in mouse pups using two-photon laser-scanning microscopy. When used together with fluorescent transgenic mouse lines and exogenous dyes that label the endothelial wall, this approach makes it possible to capture the developmental dynamics of vascular structure and NVU cell types in the intact brain of live mouse pups. Further, physiological processes including cerebral blood flow, vascular remodeling, and BBB function can be readily assessed.

There are several advantages to using two-photon imaging through chronic thinned-skull windows. First, the skull is not breached, which allows vascular development to proceed with minimal perturbation and neuroinflammation, compared with full craniotomy.^2^ Second, the approach enables visualization of morphological changes in the endothelium and the evaluation of pericyte-endothelial interaction. Although time-lapse imaging can be achieved in cultured preparations and three-dimensional (3D) organoids *in vitro*, proper development of vascular networks requires blood flow and interaction between all cells of the NVU. For example, endothelial cells cultured in isolation do not develop the same barrier properties as they do with pericyte coverage and the shear stress of blood flow.^23^ Another important benefit of this methodology is that the same structures can be imaged before and after a manipulation (e.g., stroke, drug treatment, and alongside behavioral testing) such that each animal has a baseline, reducing variability and sampling bias.

This approach also has some limitations. First, long-term imaging over many weeks is not yet possible due to bone re-growth under the cover glass. Further, skull morphology and calvarial vasculature may differ with mouse strains, requiring adaptations of this protocol.^24^ Second, dams that are raising their first litters will lead to frequent pup rejection and cannibalism. This primarily happens when surgeries are performed on the youngest pups (P0 to P4). Third, the quality of the data is in the hands of the surgeon. The window construction approach takes practice for consistency, and therefore can introduce variability into studies if multiple surgeons are involved. Fourth, imaging deeper brain structures is still not achievable even with two-photon imaging of far red fluorescent dyes. The imaging resolution achieved at depth was dependent on the age of the pup, with older pups providing better optical access. Three-photon imaging may help to overcome some of these limitations.^25^ Fifth, the imaging timeframe described coincides with mast cell development and migration,^16^ and these cells avidly uptake fluorescent dyes and obscure visibility of the pial vasculature. This makes longitudinal imaging with fluorescent i.v. dyes more challenging and requires alternation of dye colors between days of imaging.

To conclude, a window to the neonatal brain is essential for future studies of cerebrovascular development in health and disease. Simply observing the natural progression of vascular development in its native environment yields clues to how this elegant process is orchestrated by the diverse cells of the NVU and allows us to understand how disease and injury to the developing brain alter blood flow delivery and BBB development.
