# Supplementary figures and images for: Reinforced thinned-skull window for repeated imaging of the neonatal mouse brain
Source: Neurophotonics. 2022 Jun 3;9(3):031918. doi: 10.1117/1.NPh.9.3.031918 (PMC9163199; doi:10.1117/1.NPh.9.3.031918)

# Shih Lab Head Mount Holder

Material: Aluminum

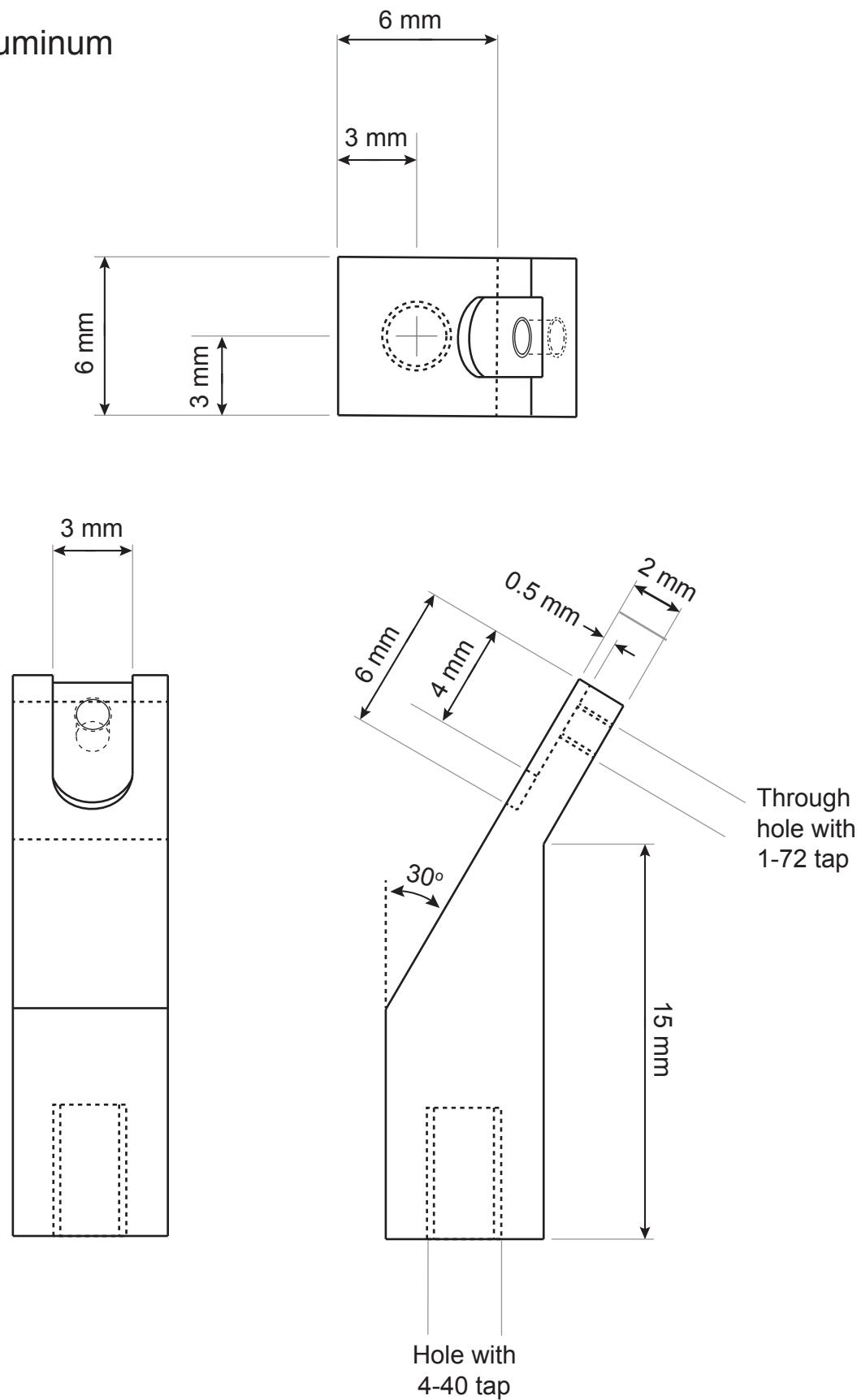

Fig S1. Coelho-Santos et al.

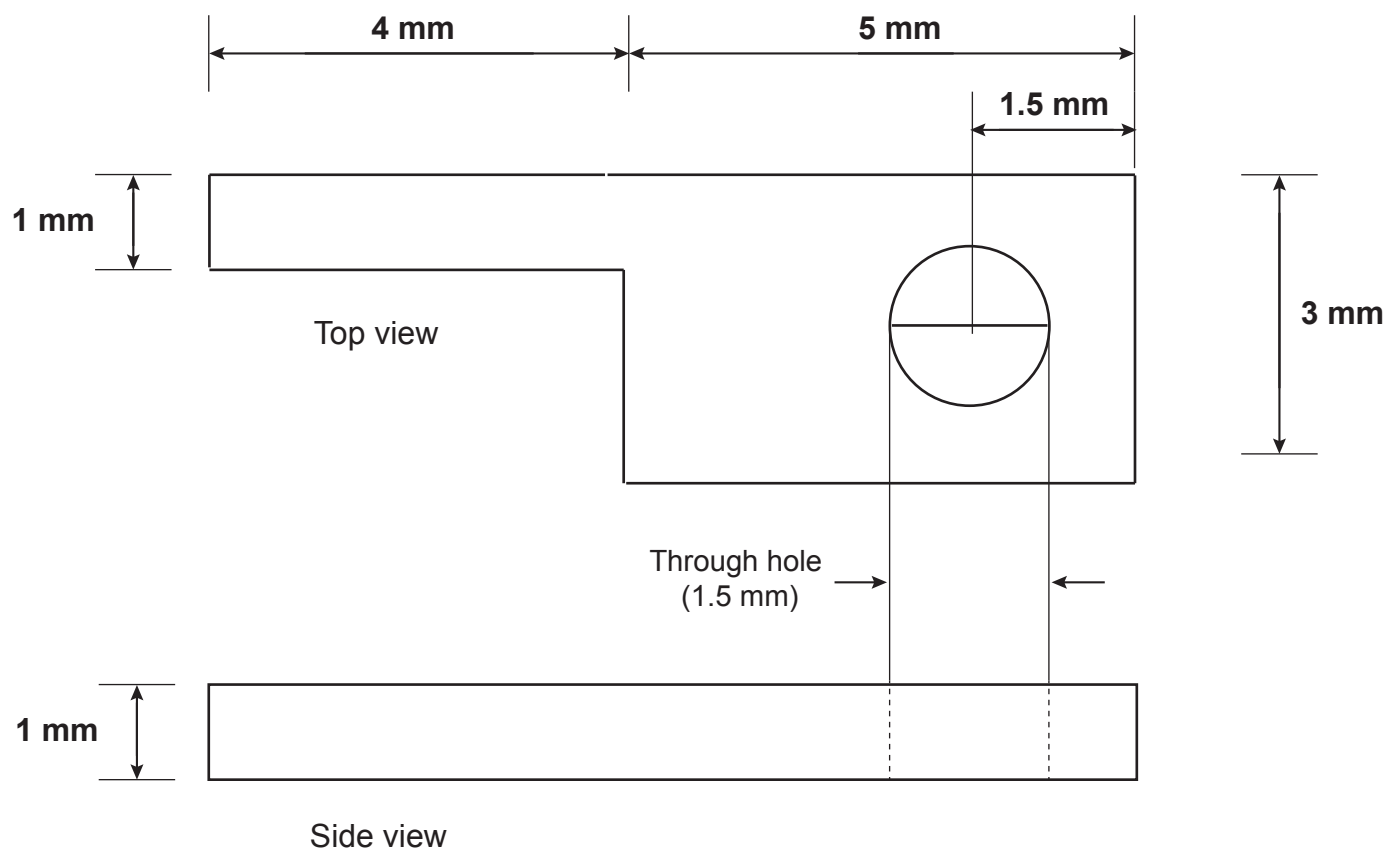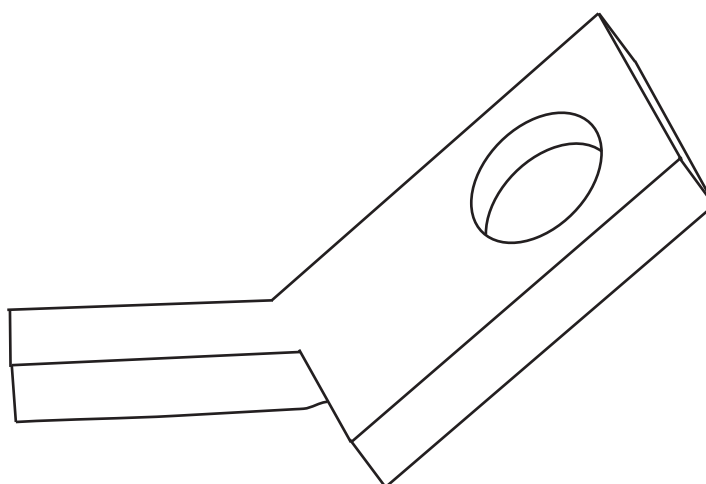

Fig S2. Coelho-Santos et al.

Supplement: Supplementary file 1 [file NPh_009_031918_SD001.pdf]
